# Advancements in gene editing technologies for probiotic-enabled disease therapy

**DOI:** 10.1016/j.isci.2024.110791

**Published:** 2024-08-22

**Authors:** Lixuan Wang, Jing Hu, Kun Li, Yuliang Zhao, Motao Zhu

**Affiliations:** 1 CAS Key Laboratory for Biomedical Effects of Nanomaterials & Nanosafety, CAS Center for Excellence in Nanoscience, National Center for Nanoscience and Technology, China, Beijing 100190, China; 2 University of Chinese Academy of Sciences, Beijing 100049, China

**Keywords:** Molecular biology, Microbiome

## Abstract

Probiotics typically refer to microorganisms that have been identified for their health benefits, and they are added to foods or supplements to promote the health of the host. A growing number of probiotic strains have been identified lately and developed into valuable regulatory pharmaceuticals for nutritional and medical applications. Gene editing technologies play a crucial role in addressing the need for safe and therapeutic probiotics in disease treatment. These technologies offer valuable assistance in comprehending the underlying mechanisms of probiotic bioactivity and in the development of advanced probiotics. This review aims to offer a comprehensive overview of gene editing technologies applied in the engineering of both traditional and next-generation probiotics. It further explores the potential for on-demand production of customized products derived from enhanced probiotics, with a particular emphasis on the future of gene editing in the development of live biotherapeutics.

## Graphical abstract

**Figure:**
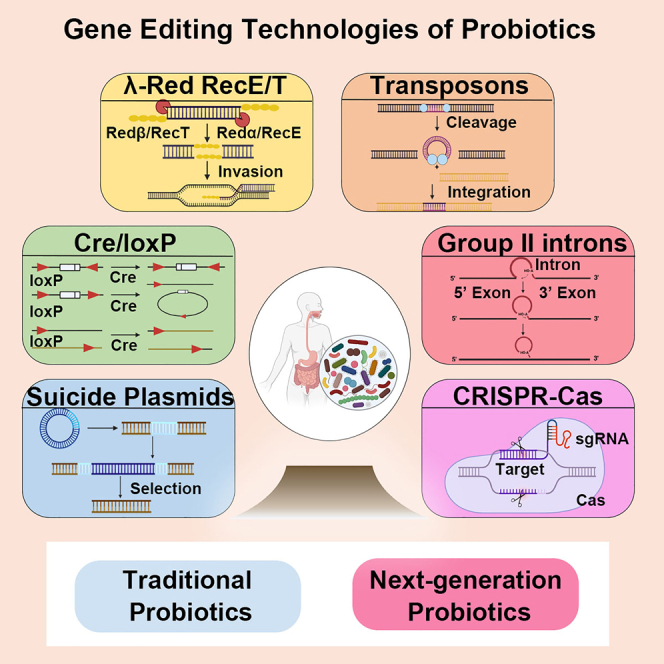

## Introduction

Probiotics are defined as “live microorganisms that, when administered in adequate amounts, confer a health benefit on the host”.^1^ The human body is naturally colonized with a number of microorganisms in the oral, digestive, and reproductive tracts. Of these, many strains present probiotic traits and are responsible for maintaining the body’s health.^2^^,^^3^^,^^4^ In addition, certain strains of probiotics are not inherently found within the human body. However, they can be introduced and supplemented into the body in a variety of ways, including through food, pharmaceutical products, and non-orally consumed products, to confer greater health benefits.^5^^,^^6^ Probiotics have been demonstrated to possess beneficial effects in the treatment of a diverse array of medical conditions. For example, supplementation with *Lactobacillus acidophilus* DDS-1 can alleviate symptoms such as bloating and diarrhea that are linked to lactose intolerance.^7^ The application of *Bifidobacterium lactis* has been found to exhibit significant preventive and therapeutic effect on irritable bowel syndrome (IBS).^8^ Furthermore, several studies have indicated that *Lactobacillus acidophilus* and *Bifidobacterium bifidum* can also enhance the immunological response of the host and enhance the functionality of immune cells.^9^^,^^10^

Despite the potential clinical applications of probiotics, the precise mechanism by which they exert their effects remains uncertain, hence impeding their integration into pharmaceutical formulations. Functional modification of probiotics allows them to perform their physiological activities while releasing active metabolites in a targeted, sustained, and controlled manner.^11^ Functional genes for disease treatment can be integrated into the genomes of probiotics, enabling the treatment of a variety of diseases such as infectious diseases,^12^ gastrointestinal disorders,^13^ and tumors.^14^ With the advent of diverse gene editing technologies, a number of probiotics have been engineered with additional functionalities. In the following sections, we will provide a concise introduction of the theoretical basis of gene editing technologies, including suicide plasmids, Cre/*loxP* system, λ-Red and RecE/T systems, transposons, group II introns, and CRISPR-Cas systems. Furthermore, we will illustrate the application of these methodologies in the genetic modification of probiotics. We will discuss the gene editing methods for both traditional and next-generation probiotics, each serving distinct objectives in the therapy of diseases. In contrast to traditional probiotics, research on next-generation probiotics is relatively limited and shallow. However, the next-generation probiotics hold potential for personalized medicine due to the ability to ameliorate specific diseases through modulating the gut microbiota. The forthcoming iteration of probiotics is considered as a rational endeavor to transition from traditional bacteria, which have a track record of safety, to novel microorganisms. This paper aims to shed information on the present state and challenges associated with gene editing in the context of probiotics, especially next-generation probiotics. The establishment of these principles will serve as the basis for the later utilization of genetically modified probiotics in the prevention and management of various diseases.

## Gene editing technologies

Gene editing technologies include a range of methodologies, such as suicide plasmids, Cre/*loxP* system, λ-Red and RecE/T systems, transposons, group II introns, and CRISPR-Cas systems. Table 1 displays the application scenarios and the advantages and disadvantages of certain gene editing technologies.

**Table 1:** Comparison of different gene editing technologies

Technologies	Application scenarios	Advantages	Defects
Suicide plasmids^15^^,^^16^^,^^17^	Deletion, insertion, and replacement of genes	Scarless editing and accessible to a broad range of hosts	Instable, high false positive rates, and complex operation
Cre/*loxP* system^18^^,^^19^^,^^20^	Deletion, inversion, and translocation of genes	Efficient, spatial and temporal specificity, no need for cofactors, and available to gene editing of large fragments and multiple recombination	Leaving scars and limited by site selection
λ-Red and RecE/T systems^21^^,^^22^^,^^23^	Deletion and insertion of genes, point mutations, and repairing DNA breaks	Scarless editing, high efficiency, and strong programmability	Dependent on homologous fragments, and not suitable for large gene fragment manipulation
Transposons^24^^,^^25^^,^^26^	Inactivation and insertion of genes, and creating mutant libraries	Efficient and flexible	Restricted insertion sites and potential interference with the functionality of the adjacent genome
Group II introns^27^^,^^28^^,^^29^	Deletion and insertion of genes	Efficient	Possible off-target integration, complex operation, and difficult in deletion or insertion of large gene fragments
CRISPR-Cas systems^30^^,^^31^^,^^32^	Deletion, insertion, and point mutations of genes	Simple, convenient, and available for large fragment editing and multiplex gene editing	Off-target effects

Abbreviations: Cre: cyclization recombination enzyme.

### Suicide plasmids

Suicide plasmids can be transferred between bacteria through conjugation, a process where two bacteria form a physical connection and exchange genetic material.^33^ They are often used to create knockout mutants in Gram-negative bacteria.^34^ Suicide plasmids are designed to have limited or no ability to replicate in the recipient bacteria, ensuring that they do not persist after facilitating genetic modification, thus preventing unwanted effects.^35^

**Figure 1:**
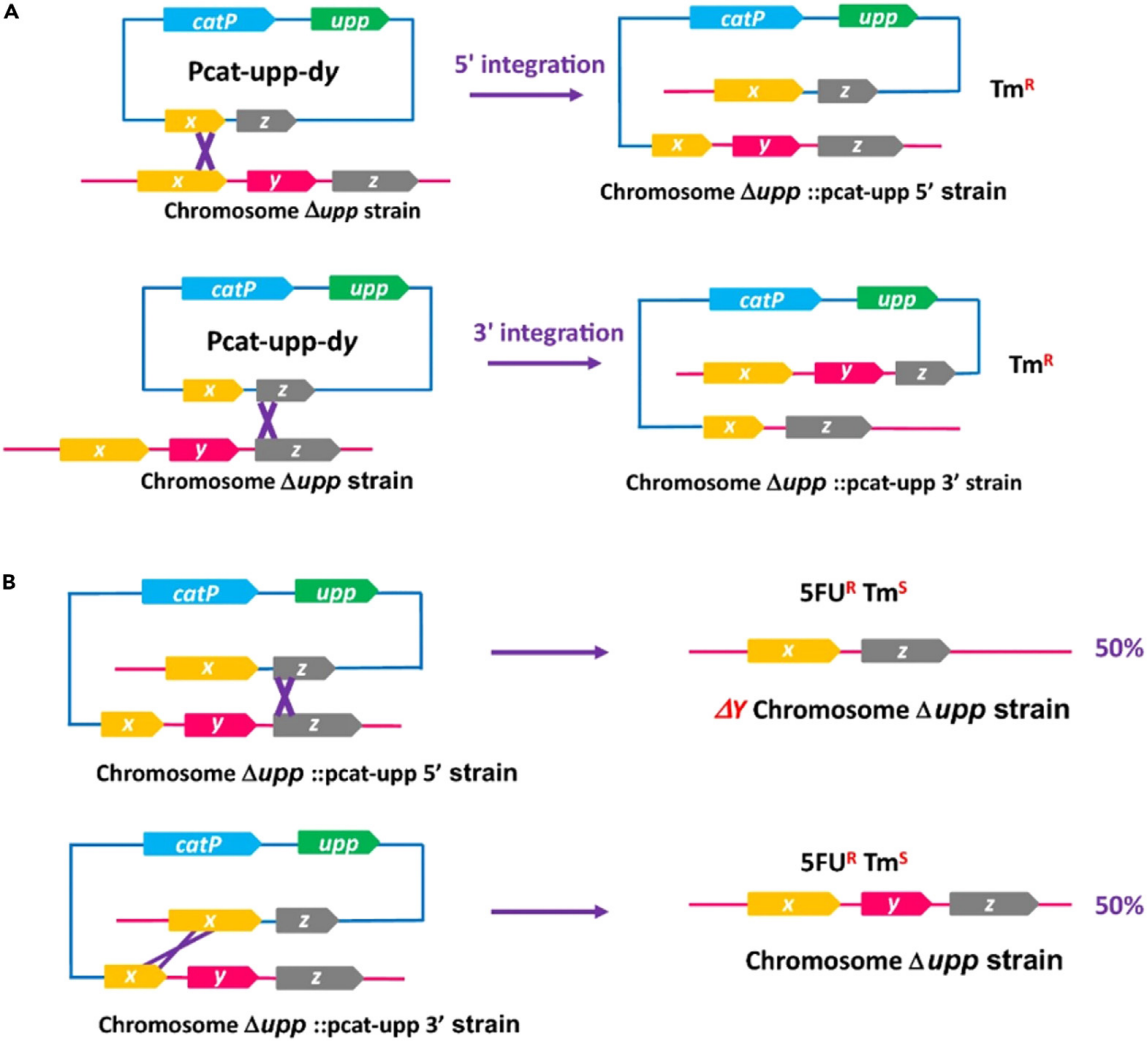
Scarless gene deletion in *Clostridium* by suicide plasmids combined with the counterselection system The use of restrictionless Δ*upp* strains and suicide vectors containing an antibiotic resistance gene and *upp* allowed for the achievement of scarless gene editing in *Clostridium*. (A) The suicide plasmid can be integrated into the host bacterial genome in either the 5′ or 3′ direction through homologous recombination, utilizing antibiotic resistance for the selection of the first crossing-over. (B) The double crossing-over and the excision of the plasmid can be achieved by employing the 5-FU/*upp* system. *X* and *z* genes represent the homologous regions in the suicide plasmid, and *y* gene is to be deleted. Reprinted with permission from [Foulquier et al.^42^]. Copyright (2019) Springer Nature. Abbreviations: *upp*: gene that encodes uracil phosphoribosyl-transferase (UPRTase), 5-FU: 5-fluorouracil.

A two-stage homologous recombination process is involved in the application of suicide plasmids for gene editing. The initial step involves integrating suicide plasmids into the bacterial genome, which is typically screened by antibiotic resistance. The subsequent step is to remove the backbone of suicide plasmids from the bacterial genome, and the screening of genetically engineered bacterial mutants is mainly achieved by counterselection markers.^15^ The *sacB* gene encodes a levansucrase that can inhibit most Gram-negative bacteria in sucrose medium, so integration of the *sacB* gene into suicide plasmids allows screening of gene-edited bacteria.^36^ However, the implementation of counter-selection methods using the *sacB* gene results in a high false positive rate.^37^^,^^38^ The false positive rate of the *upp* gene counterselection system is approximately 10 times lower than that of the *sacB* counterselection system,^37^ so it is commonly used for a variety of probiotics.^39^ The *upp* gene encodes for uracil phosphoribosyl transferase (UPRTase), an enzyme that converts 5-fluorouracil (5-FU) to 5-fluoro-UMP that inhibits thymidylate synthase activity, ultimately leading to cell death.^40^^,^^41^ Only cells lacking the *upp* gene can survive in the presence of 5-FU. The process of gene editing in *Clostridium* by suicide plasmid vectors combined with *upp* counterselection marker is shown in Figure 1.^42^

The application of suicide plasmids-mediated gene editing technology can achieve scarless editing of target genes and edit gene fragments at most locations in the bacterial genome.^43^ However, this technology requires suicide plasmids to be transferred into the host bacteria and bound to the chromosome, which may introduce instability to the gene editing process.^44^

### Cre/*loxP* system

Sternberg and Hamilton first described a recombinase from P1 phage and named it cyclization recombination enzyme (Cre).^45^ Cre is a recombinase that recognizes specific *loxP* sites that are 34 bp in length and consists of an asymmetric 8 bp spacer flanked by 13 bp of inverted repeats.^46^ Recombination of two *loxP* sites in the same orientation results in the deletion of the flanked DNA segment. Conversely, recombination of two *loxP* sites in the opposite orientation results in the inversion of the *loxP*-flanked DNA segment. Cre recombinase also catalyzes translocation when *loxP* sites are present on two different chromosomes.^47^

The advantage of Cre recombinase is that they do not require any cofactors to function, so that Cre/*loxP* recombination system is broad-spectrum and highly active, and theoretically, can effectively work on any type of DNA in any cellular environment. Compared to CRISPR-Cas systems, it neither produces DNA double-strand breaks (DSBs), nor relies on the natural homology-directed repair pathway of bacteria. Cre/*loxP* recombination system can achieve large DNA segment deletions of more than 25 kb and allow multiple recombination at the same time.^48^ In bacterial gene editing, Cre/*loxP* recombination system can be used to delete selective genes for identifying the function of dextranase on sugar metabolism of *Leuconostoc mesenteroides* DRP105.^49^ Notably, site-specific recombination strategies with high efficiency and precise spatiotemporal resolution are continuously being developed. In addition to the common chemical induction, Cre/*loxP* recombination system based on optical induction are also constantly being investigated for more precise gene editing.^50^^,^^51^

**Figure 2:**
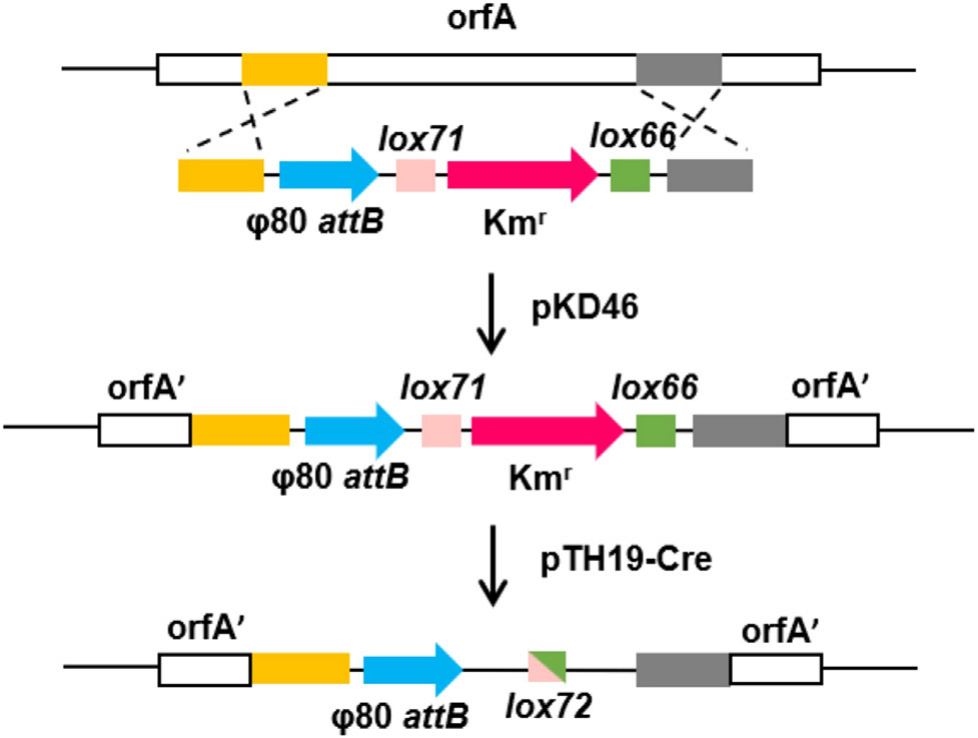
Diagram of the insertion of the additional ϕ80 *attB* site on the EcN chromosome using Cre/lox72 system The additional ϕ80 *attB* site was inserted in orfA on the EcN chromosome by double homologous recombination assisted by the helper plasmid pKD46. After insertion, the selection marker Km^r^ flanking the *lox66* and *lox71* sites was removed by the helper plasmid pTH19-Cre, and *lox72*, the recombination site for *lox66* and *lox71*, was formed. Modified and reprinted with permission from [Cheng et al.^56^]. Copyright (2022) American Chemical Society. Abbreviations: ϕ80 *attB* site: ϕ80 bacterial attachment site, Km^r^: kanamycin resistance gene, *lox66*: mutant *loxP* sites, *lox71*: mutant *loxP* sites, *lox72*: recombination site of *lox66* and *lox71*.

Repeated use of natural *loxP* sites for gene replacement and selectable marker removal can result in the accumulation of multiple *loxP* sites capable of being recognized by Cre recombinase in the genome, which cause genetic instability.^52^ To address this issue, scientists have altered the *loxP* site to produce a mutant left element (*lox66*) and a mutant right element (*lox71*), which can still be targeted by Cre recombinase. When *lox66* and *lox71* combine, they create a novel *lox72* site, which will not receive recognition and action from Cre recombinase like the original *loxP* site. Therefore, its binding affinity is significantly reduced, allowing for multiplex gene deletion operations without introducing additional genetic instability.^53^ The modified Cre/lox system has been successfully used for large fragment gene deletion in *Lactobacillus*^54^ and *Bacillus subtilis*^55^ and EcN. The insertion efficiency in EcN for multiple phage attachment site integration was significantly improved to about 37–61%^56^ (as shown in Figure 2).

### λ-Red and RecE/T systems

The recombination system using λ-Red and RecE/T requires only short homologous DNA fragments (50 bp) for efficient homologous recombination to enable knockout, insertions and point mutations in the bacterial genome.^57^

**Figure 3:**
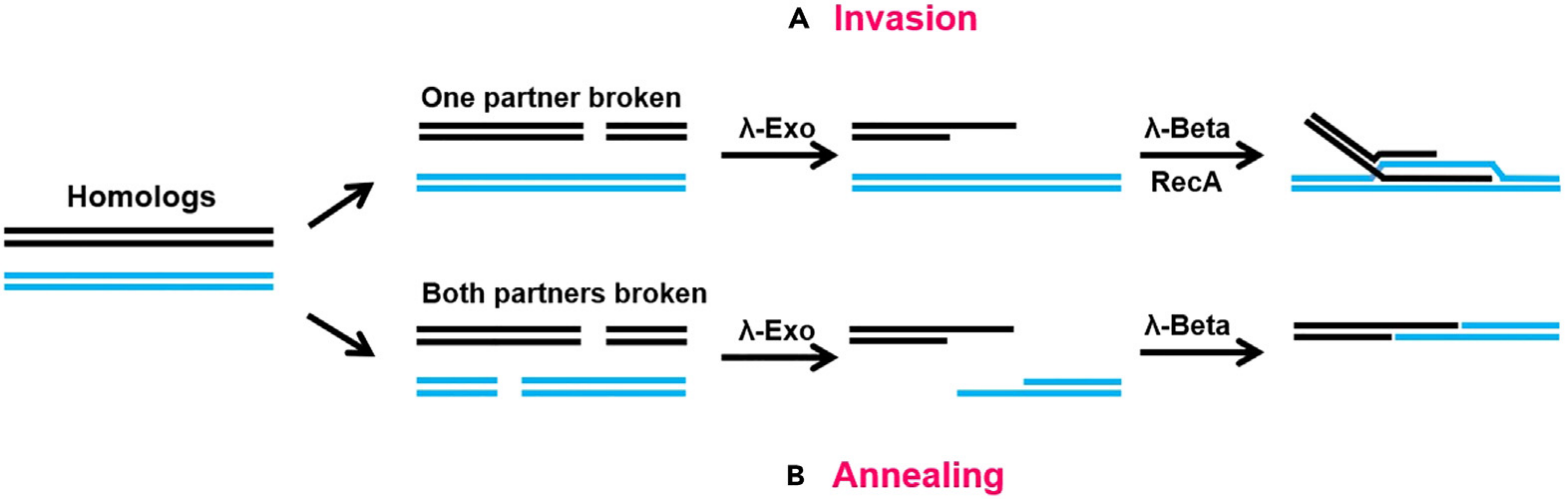
Homologous recombination process mediated by λ-Red system The DSB repair process mediated by λ-Red system is primarily divided into two different mechanisms. λ-Exo cleaves dsDNA to produce a 3′ overhang in both pathways. (A) Invasion: When the recombination partner with the dsDNA end is an unbroken circular homologous duplex, the recombination process will proceed through strand invasion dependent on RecA protein. (B) Annealing: When the recombination partner is replicating ssDNA, the recombination process will be facilitated through annealing mediated by λ-Beta. Modified and reprinted with permission from [Poteete et al.^60^]. Copyright (2001) Federation of European Microbiological Societies. Abbreviations: DSBs: double-strand breaks, dsDNA: double-stranded DNA, ssDNA: single-stranded DNA.

λ-Red recombination system is derived from three proteins, λ-Gam, λ-Exo and λ-Beta, expressed in the λ-phage red motif, which are involved in homologous recombination of the genome with exogenous genes.^58^ λ-Gam can inhibit intracellular nucleic acid exonucleases and endonucleases to prevent degradation of exogenous genes. λ-Exo, as a nucleic acid exonuclease, is able to degrade exogenous genes along the 5′-3′ direction, resulting in single-stranded gaps at both ends of exogenous double-stranded DNA (dsDNA). λ-Beta promotes annealing pairing of exogenous genes with homologous sequences in the genome.^59^ λ-Red homologous recombination process involves two mechanisms: invasion and annealing^60^ (as shown in Figure 3). The invasion process typically occurs when only one partner is broken. λ-Exo cleaves dsDNA to produce 3′ ends, and RecA allows the single-stranded DNA (ssDNA) to enter the homologous double strand. Subsequently, the homologous recombination process is completed through branch migration and Holliday junctions. If two partners are broken, the annealing process is triggered. After λ-Exo cleavage of dsDNA, λ-Beta facilitates the annealing process and recombinants are formed by ligating.^61^^,^^62^ Antibiotic resistance genes are commonly utilized as selectable markers and later removed through plasmids expressing flippase recombination enzyme or Cre recombinase (for *loxP*), or by a second recombination with unlabeled linear DNA.^63^ λ-Red recombination process had a recombination efficiency of 0.2% with dsDNA and up to 25% with ssDNA.^64^ λ-Red recombination system is capable of accomplishing efficient integration of 2000 bp DNA fragments, but for large DNA fragments (>2000 bp), the recombination efficiency decreases dramatically.^65^

RecE/T recombination system, derived from the *E. coli* Rec phage, is similar to λ-Red recombination system. RecE acts as a nucleic acid exonuclease, excising linear DNA to expose ssDNA tails, while RecT binds to ssDNA and facilitates the annealing pairing of ssDNA to the genome.^66^ This recombination system is often coupled with CRISPR-Cas systems to recombinantly repair the cleavage site after DSBs.^67^ The RecE/T recombination-assisted CRISPR-Cas9 gene editing technology increases the frequency of double-exchange events at the chromosomal DSBs and between repair templates in *Corynebacterium glutamicum*.^68^

### Transposons

Transposons are mobile genetic elements that can move independently from one location to another in the genome.^69^ Transposon random mutagenesis is an established technology for building libraries of mutant bacterial strains. Mutants can be obtained by inserting transposons into genes and directly disrupting the reading frame, which is a rapid and simple process.^69^ Several types of transposons have been used to construct single gene mutant libraries in bacteria, such as Tn3,^70^ Tn5,^71^ Tn7,^72^ and mariner family transposable elements.^73^ Himar1 is the most commonly used transposable element of the mariner family in bacterial mutagenesis. It catalyzes the insertion of TA dinucleotide sites with high randomness and coverage, allowing for the construction of a library of bacterial transposon random insertion mutants.^74^ Himar1 transposable element has been successfully constructed as a high-throughput mutagenesis system in *Bacteroides*.^75^ Recently, it has been shown that CRISPR-Cas systems encoded in transposons can mediate RNA-directed site-specific transposition, enabling efficient and specific insertion of exogenous genes into the genome. This technology is known as CRISPR-associated transposase from cyanobacteria *Scytonema hofmanni* (ShCAST) and has been successfully used for precise insertion into the *E. coli* genome.^76^^,^^77^

### Group II introns

Group II introns, known as retrotransposons, are site-specific reverse transcription factors with nuclease activity.^78^ The secondary structure of group II introns consists mainly of six stem-loop structural domains distributed in a radial pattern, and this structure enables them to catalyze their own splicing reactions.^79^ During target site recognition, the exon binding sequence (EBS) of group II introns recognizes the intronic binding sequence (IBS) of the target site by base complementary pairing. After recognition, group II introns cleave the target site DNA sense strand by reverse splicing, and the host DNA repair mechanism is utilized to form a complete DNA double strand. This process is called “Retrohoming”.^80^^,^^81^

**Figure 4:**
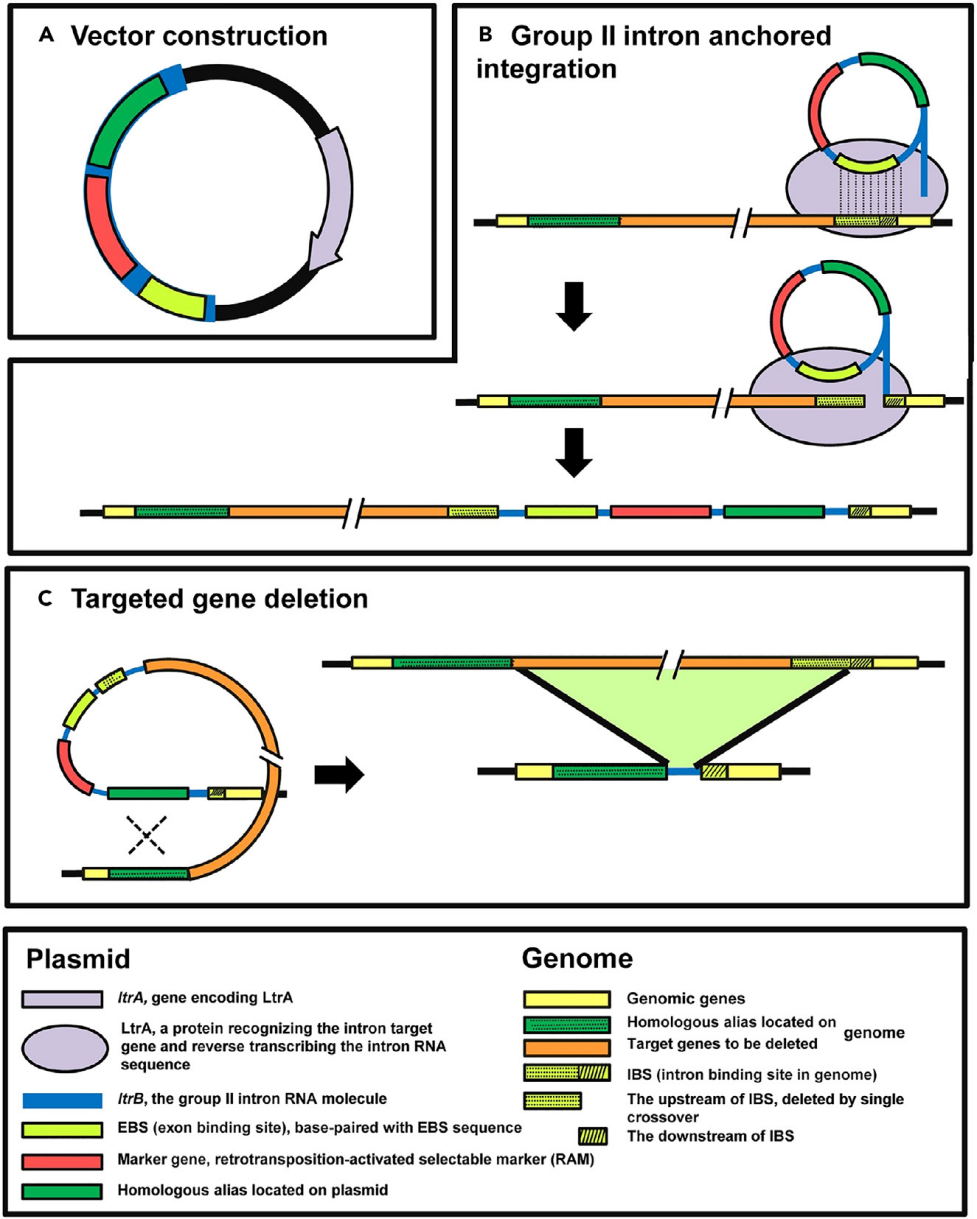
Intron-anchored gene deletion approach in *Clostridium* (A) Vector structure containing Ll. ltrB introns. (B) The EBS of Ll. ltrB introns matched the IBS on the target gene and underwent reverse transcription catalyzed by LtrA. The Ll. ltrB introns were inserted into the target gene. (C) Deletion of the target gene by homologous recombination, and no antibiotic resistance markers were left in the genome during the gene deletion process, which facilitated subsequent gene manipulation. Reprinted with permission from [Jia et al.^84^]. Copyright (2011) PLOS ONE. Abbreviations: EBS: exon binding sequence, IBS: intronic binding sequence, LtrA: protein that recognizes the intron target gene and reverse transcribes the intron RNA sequence.

Based on the RNA structure of introns, group II introns are mainly classified into three categories (IIA, IIB, and IIC).^82^ Among them, the group IIA intron Ll. ltrB from *Lactococcus lactis* is one of the most studied and widely used group II introns. Based on the target site recognition properties of Ll. ltrB introns and their migration patterns on chromosomes, it has been developed as an efficient gene targeting tool, Targetron.^83^ Similarly, the general gene targeting tool specifically used for *Clostridium* is called ClosTron^84^ (as shown in Figure 4).

Some group II introns without intron-encoded protein (IEP) domains were also found to have significant cleavage activity, named as hydrolytic endonucleolytic ribozymes (HYERs). Recent studies have shown that HYERs can exhibit DNA-cutting efficiency *in vitro* comparable to CRISPR-Cas12 system. Compared to CRISPR-Cas systems, HYERs (0.6 kb) are much more compact than the CRISPR nuclease (3–4 kb).^85^ More importantly, group II introns also work for bacteria lacking homologous recombination and can be modified to integrate into any locus of interest, expanding the means of gene editing for microorganisms that are inefficient at transforming exogenous DNA.^86^ However, group II introns-mediated gene editing is to realize the inactivation of target genes through the insertion of introns at the target site, and thus cannot realize functions such as point mutation and scarless knockout of genes. Besides, due to the limited size of the genes it carries, the insertion of large fragments and multiple genes cannot be realized.^27^^,^^84^

### CRISPR-Cas systems

**Figure 5:**
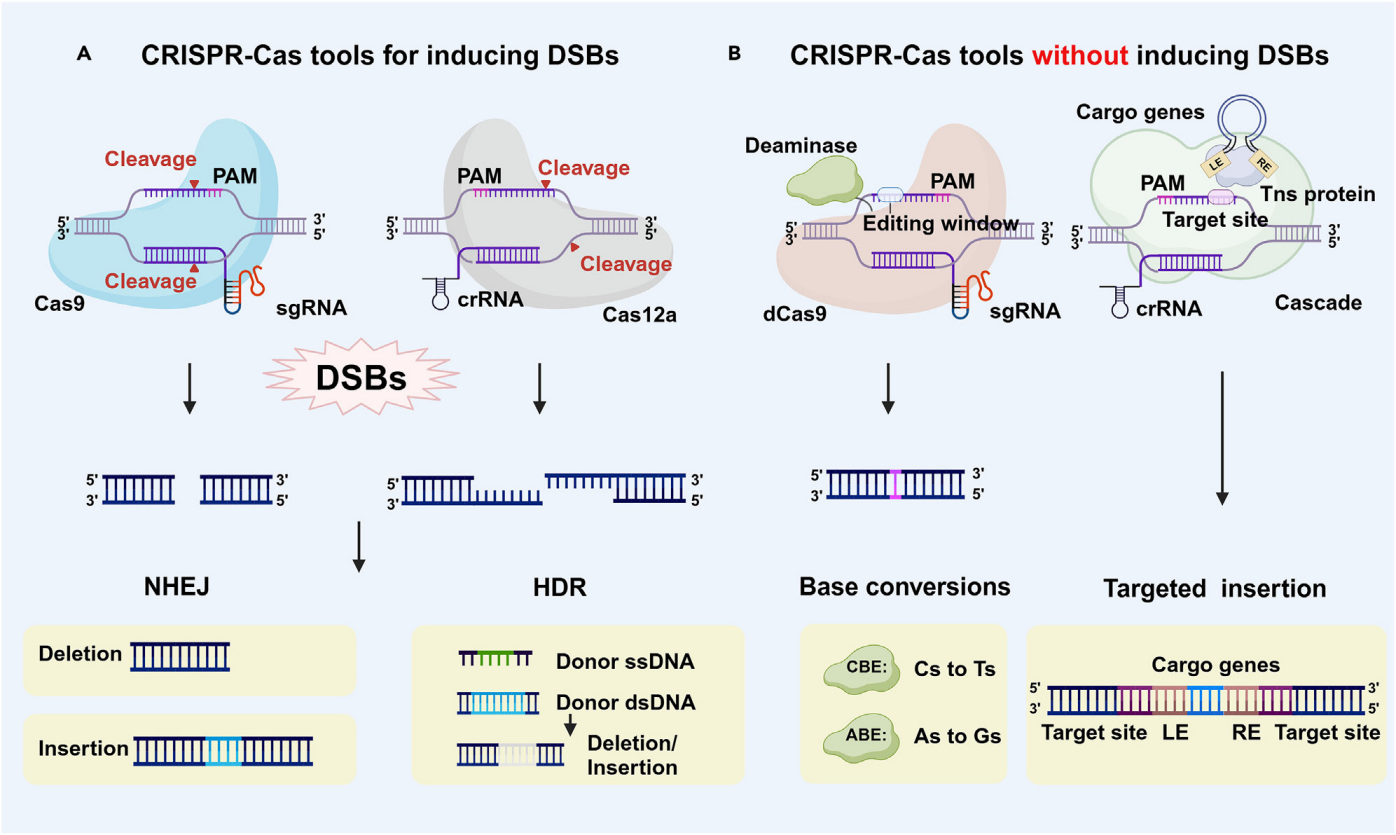
Diagram of several CRISPR-Cas-mediated gene editing processes (A) CRISPR-Cas tools capable of generating DSBs. Cas9 cleaves DNA to create blunt ends under the direction of sgRNA while Cas12a (Cpf1) cleaves DNA with the assistance of crRNA to produce sticky ends. After the formation of DSBs, recombination repair process is accomplished through NHEJ or HDR, resulting in gene insertion or deletion. (B) CRISPR-Cas tools that do not generate DSBs. CRISPR-dCas9 system coupled with the base editor enable single-base mutations without DSBs. Bacterial CASTs facilitate the insertion of targeted DNA sequences without the generation of DSBs. Created with BioRender.com. Abbreviations: sgRNA: small guide RNA, crRNA: CRISPR RNA, PAM: protospacer adjacent motif, dCas9: a mutant form of the Cas9 protein, Tns: transposons, DSBs: double-strand breaks, NHEJ: non-homologous end-joining, HDR: homology directed repair, CBE: cytosine base editor, C: cytosine, T: thymine, ABE: adenine base editor, A: adenine, G: guanine, LE: left end, RE: right end, CASTs: CRISPR-associated transposases.

CRISPR-Cas systems, as the 3rd generation genome editing technology, have multiple advantages including simple design, outstanding editing efficiency, and high specificity compared with traditional Zinc-finger nucleases (ZFN) and Transcription activator-like effector nucleases (TALEN) technologies.^87^ ZFN recognizes specific nucleic acid sequences through zinc finger structure and cuts DNA through Fok Ⅰ nucleic acid endonuclease activity.^88^ TALEN recognizes specific nucleic acid sequences through transcriptional activation-like effector and cuts DNA through Fok Ⅰ nucleic acid endonuclease activity.^89^ Comparatively, CRISPR-Cas systems recognize specific nucleic acid sequences through small guide RNA (sgRNA) and cut DNA through Cas protein.^90^ Several common CRISPR-Cas mediated-gene editing processes are shown in Figure 5. ZFN and TALEN are more widely used for gene editing in eukaryotic cells. By contrast, CRISPR is discovered from prokaryotic organisms and therefore facilitates its application in bacteria gene editing.^91^

CRISPR-Cas systems can be classified into two categories of six types: the first category uses effector complexes composed of multiunit Cas protein and mature CRISPR RNA (crRNA) to recognize and cleave target nucleic acids, including type I, III and IV; the second category functions through effector complexes composed of individual Cas protein and crRNA, including types II, V, and VI.^92^ Type I systems account for the largest number of CRISPR-Cas systems, and the Cas3 protein can be used as the characteristic protein. Type III systems not only have the ability to resist invading DNA, but also show resistance to invading RNA, and the Cas7-11 proteins are the signature proteins. Many of type IV systems lack nuclease proteins, but nucleic acids from different plasmids can be detected in their spacer region sequences, so type IV systems may be related to resistance to foreign plasmids. The representative of type II systems is CRISPR-Cas9 system, and the Cas9 proteins currently used are mainly derived from *Streptococcus pyogenes* (SpCas9) and *Staphylococcus aureus* (SaCas9). Due to its relatively common protospacer adjacent motif (PAM) sequence and simple and rapid editing process, it has become the most widely used technology in CRISPR-Cas systems. The most widely used type V system is CRISPR-Cas12a (Cpf1) system, among which *Acidaminococcus* sp. BV3L6 (AsCas12a), *Francisella novicida* U112 (FnCas12a) are often developed as genome editing tools for prokaryotic and eukaryotic cells. The Cas9 protein leaves flat DNA ends after cutting dsDNA, while the Cas12a (Cpf1) protein leaves sticky ends, a structure that facilitates non-homologous end-joining (NHEJ) during gene repair process, allowing for more precise gene insertion.^93^^,^^94^ The Cas13 protein of type VI system is an RNA-guided RNA endonuclease.^95^

Many studies have shown that CRISPR systems tend to achieve editing of bacterial genomes in two ways: either by using bacterial endogenous CRISPR for editing, or by introducing exogenous CRISPR-Cas systems. There have been various examples of bacteria utilizing endogenous CRISPR-Cas mechanisms to achieve gene editing. For example, multiplex gene editing was achieved using the type I-B system on the genome of *Clostridium tyrobutyricum* to improve the production of butanol,^96^ and the type II-A system can be applied to gene deletion, gene insertion, and point mutation in *Pediococcus acidilactici*.^97^ However, since homology directed repair (HDR) activity in bacteria is typically low and the NHEJ system is present in only a few bacterial species, such as *Firmicutes*, *Proteobacteria*, and *Actinobacteria*,^98^^,^^99^ they need to be provided with homologous recombination templates for repairing DSBs after cleavage by Cas proteins.

The complexity of bacterial genomes and the fact that different bacteria may exhibit disparate adaptations to distinct gene editing methodologies render the application of a single gene editing technology an inefficient approach. The integration of CRISPR-Cas systems with other technologies can significantly expand the scope of applications and enhance the efficacy of editing.^35^^,^^100^^,^^101^ For example, CRISPR-Cas9 system can increase the accessibility and flexibility of gene editing when coupled with Cre-lox system. After the integration of the vector carrying expression cassettes into the host chromosome, Cre recombinase removes the inserted replicon and selection markers flanking *lox66* and *lox71*. CRISPR-Cas9 system then cleaves the expression cassettes to restore the original function when they are no longer required.^102^ CRISPR-Cas12a (Cpf1) system can be employed in combination with RecE/T system. The crRNA is capable of inducing the Cas12a (Cpf1) nuclease to cleave the bacterial genome, thereby generating DSBs. RecE/T system can assist the recombination repair process of ssDNA and dsDNA by the RecE and RecT proteins, which ultimately leads to the efficient gene editing.^67^ Additionally, Type V-K CRISPR-Cas system has been demonstrated to function effectively in conjunction with transposons. The helper plasmid (pHelper) contains four CRISPR-associated transposases (CAST) genes (*tnsB*, *tnsC*, *tniQ*, and *Cas12k*) and crRNA genes targeting a synthetic PSP1. The donor plasmid (pDonor) connects the targeting sequences to be inserted in the middle of the transposon left-end and right-end. The target plasmid (pTarget) carries the PAM and PSP1 sequences. By transforming the three plasmids into bacteria, the genes could be targeted for insertion between 60 and 66 bp at the 3′ end of PAM. The ShCAST system was shown to enable the insertion of up to 10 kb of DNA in bacteria.^103^ Furthermore, CRISPR-dCas9 system can be utilized in conjunction with base editors. dCas9 is a specific mutation at the cutting site of the Cas9 protein that renders it inactive as a nucleic acid endonuclease, which enables precise localization and binding to the target site under the guidance of sgRNA. Base editors enable the conversion of cytosine to thymine or adenine to guanine.^32^ The examples and advantages of CRISPR-Cas systems combined with other technologies are shown in Table 2.

**Table 2:** Examples and advantages of integrating CRISPR-Cas systems with other technologies

CRISPR-Cas systems	Jointly employed technologies	Examples	Advantages
CRISPR-Cas9 system^102^	Cre/lox system	After integrating the vector carrying expression cassettes into the host chromosome, Cre recombinase removes the inserted replicon and selection markers flanking *lox66* and *lox71*. CRISPR-Cas9 system cleaves the expression cassettes to restore the original function when they are no longer needed.	Enhancing the versatility and flexibility of gene editing while minimizing the error rate.
CRISPR-Cas12a (Cpf1) system^67^	RecE/T system	The genome is cleaved by crRNA-directed Cas12a (Cpf1), resulting in DSBs. These DSBs are then repaired using the RecE/T recombination system, which involves both ssDNA and dsDNA recombination.	Increasing homologous recombination activity and achieving robust expression of Cas12a (Cpf1). Enabling the editing of multiple genes and large DNA fragments.
Type V-K CRISPR-Cas system^103^	Transposons	Cas12k recognizes specific sequences, and Tn7-like transposons insert exogenous gene fragments into the genome.	Achieving efficient and specific insertion of large DNA fragments without inducing DSBs and is independent of homologous recombination repair process.
CRISPR-dCas9 system^32^	Base editors	Multiple sgRNAs are used to localize the genome of *Bacillus subtilis*, while CBEs are employed to achieve cytosine to thymine base-shift mutations within the target site activity window.	Improving multiplex gene editing efficiency without inducing DSBs and is independent of homologous recombination repair process.

Abbreviations: Cre: cyclization recombination enzyme, crRNA: CRISPR RNA, DSBs: double-strand breaks, ssDNA: single-stranded DNA, dsDNA: double-stranded DNA, dCas9: a mutant form of the Cas9 protein, sgRNA: small guide RNA.

**Figure 6:**
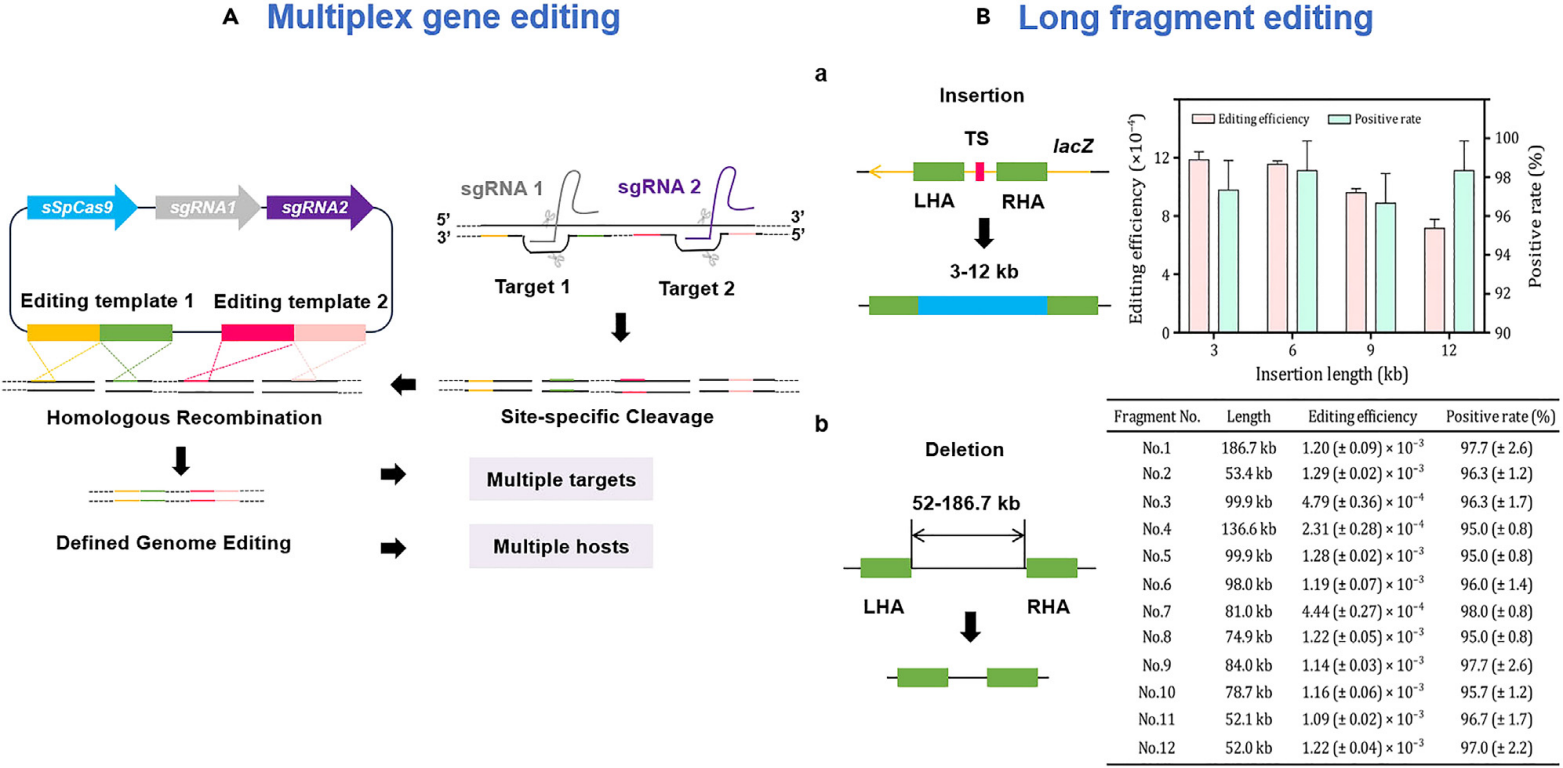
Advanced gene editing functions enabled by CRISPR-Cas systems (A) Multiplex gene editing can be achieved using CRISPR-Cas9 system, which involves the use of the same Cas9 endonuclease and two sgRNA cassettes. Modified and reprinted with permission from [Cobb et al.^107^]. Copyright (2014) American Chemical Society. (B) Insertion and deletion of long DNA fragments can be achieved by combining the use of CRISPR-Cas9 system and λ-Red recombination system. (a) The insertion of gene fragments ranging in length from 3 to 12 kb into the *lacZ* gene resulted in positive rates above 95% in all cases. (b) The deletion of 12 long fragments ranging in length from 52.0 to 186.7 kb, all with a positive gene editing rate of more than 95%. Modified and reprinted with permission from [106]. Copyright (2020) Springer Nature. Abbreviations: TS: target site, LHA: left homologous arm, RHA: right homologous arm.

There are still many difficulties with multiplex gene editing as well as large fragment gene editing in bacteria. For instance, simultaneous gene editing of multiple loci is less efficient and prone to off-target effects.^104^ Gene editing of large segments frequently results in inefficiencies and complex repair mechanisms.^64^ CRISPR-Cas systems serve as promising gene editing tools for advanced gene editing functions. For multiplex gene editing, different specific gRNAs are designed to guide Cas proteins to reach multiple target gene sites and cut the target sequences. The process of gene editing is then achieved through various DNA repair mechanisms.^64^ For long fragment editing, CRISPR-Cas systems can also generate DSBs by Cas protein cleavage, and then homologous recombination can be used to accurately and efficiently edit large gene fragments. Furthermore, the editing efficiency of large fragment gene editing can be enhanced by the utilization of the recently reported CRISPR-Cas12a (Cpf1) system,^105^ or by the combination of CRISPR-Cas systems with the λ-red recombination system^106^ or transposase.^77^ The process of multiplex gene editing and large fragment editing using CRISPR-Cas systems is shown in Figure 6.

## Approaches and applications of gene editing in probiotics

The primary objectives accomplished by means of gene editing traditional and advanced probiotics encompass comprehending their processes of operation and augmenting advantageous functionalities.

### Traditional probiotics

Many gene editing technologies have been successfully applied to a variety of traditional probiotics such as *Lactobacillus*, EcN, *Bacillus subtilis*, *Bifidobacteria*, and *Saccharomyces*, and the specific technical features and application scenarios are shown in Table 3.

**Table 3:** List of successfully engineered traditional probiotics using different gene editing technologies

Bacteria	Technologies	Features & Applications
*Lactobacillus*	Double-crossover recombination	Inefficiency of vector excision, and complexity of operation^108^^,^^109^
	Cre/lox recombination system	Constructing a multigene deletion system with 99.4% correctness^53^
	DsDNA recombination mediated by λ-Red and RecE/T systems	Improving the efficiency of gene editing, but scarring when removing selection markers^110^^,^^111^
	SsDNA recombination assisted by ssDNA binding proteins	Increasing the efficiency, but only suitable for point mutations and short fragment deletions^112^
	CRISPR-Cas9 system	Knocking out mucin genes for elucidating the mechanism of gut attachment and colonization^113^
	CRISPR-Cas9 system	Knocking out the PyrR gene and revealing the essential role of PyrR in the inhibition of pathogen growth^114^
	DsDNA and ssDNA recombination assisted by CRISPR-Cas9 system	Increasing the production of GlcNAc^115^
	Suicide plasmids carrying the I-SceI recognition site	Realizing the integration of GOI genes into the EcN genome markerlessly and scarlessly^116^
EcN	λ-Red recombination system	Enabling T7-promoter EcN to produce heme proteins and heme-derived molecules^117^
	RecA recombination system	Increasing the production of heparosan^118^
	CRISPR-Cas9 system	Knocking out two cryptic plasmids to increase the production of GABA to treat hypertension, epilepsy, and anxiety^119^
	CRISPR-Cas9 system	Integrating the gene fragment of GLP-1 into the *attB* site of the EcN genome to increase the secretion of GLP-1 and exerting neuroprotective effects against PD^120^
	CRISPR-Cas9 system	Fusing the EcN outer membrane protein OmpF with MPER in HIV-1 to induce the immune response against HIV-1^121^
	λ-Red recombination system and Cre/*loxP* system	Knocking out the *myc* gene cluster, but with low editing efficiency^122^
*Bacillus subtilis*	SsDNA recombination assisted by GP35	Increasing the recombination frequency, but dependent on ssDNA and long homologous arms (500 bp)^123^
	5′-phosphorothioate modified dsDNA recombination	Allowing efficient knockout and knock-in^124^
	Red/ET recombination system	Realizing the heterologous expression of large-band gene clusters^125^
	CRISPR-Cas9 system	Gene editing using a single plasmid system^126^
	Combining CRISPR-dCas9 system with cytidine deaminase	Overcoming the challenge of low efficiency of transformation and multiplex gene editing^32^
	CRISPR-Cas9n system	Improving the efficiency of multiplex gene editing and the deletion of large fragments Liu et al.^127^
	CRISPR-Cas12a (Cpf1) system	Improving the efficiency of multiplex gene editing^128^
	Suicide plasmids	Enabling efficient gene editing independent of transformation efficiency^129^
*Bifidobacteria*	Endogenous I-G CRISPR-Cas system in combination with an exogenous CRISPR base editor	Overcoming the difficulty in genetic manipulation^130^
	CRISPR-δ integration	Increasing the copy number of the integrated genes, resulting in the activity of CMCase to 559 U/L^131^
*Saccharomyces*	CRISPR-Cas system	Integrating large DNA fragments into the yeast chromosome, resulting in the production of BDO^132^

Abbreviations: PyrR: pyrimidine regulatory gene, GlcNAc: N-acetylglucosamine, GOI: gastrointestinal gene of interest, GABA: Gamma-aminobutyric acid, GLP: glucagon-like peptide, CMCase: carboxymethyl cellulase, BDO: (*R*, *R*)-2, 3-butanediol.

### Lactobacillus

*Lactobacillus* was first evident to exert health benefits in 1907, and since then *Lactobacillus* has been increasingly supplemented in food for gastrointestinal health.^133^ *Lactobacillus* is a Gram-positive bacterium that is primarily fermentative and ultimately metabolizes to produce lactic acid.^134^ *Lactobacillus* normally lives in carbohydrate-rich environment and colonizes mammalian mucosal surfaces such as the oral cavity, gastrointestinal tract and vagina, where it plays a role in gut immunity and epithelial barrier stabilization.^135^ Within the *Lactobacillus* taxon, several strains of *Lacticaseibacillus casei*, *Lactobacillus johnsonii*, *Lacticaseibacillus rhamnosus*, and *Lactiplantibacillus plantarum* have been approved as probiotics for incorporation into foods and pharmaceuticals that confer benefits to human health.^136^^,^^137^

The initial attempt to gene editing in *Lactobacillus* was through homologous recombination using a vector-based double-crossover strategy.^138^^,^^139^ But this approach is limited by the inefficiency of vector excision and the complexity of operation.^109^ Therefore, the site-specific Cre/lox recombination system was developed and allowed the construction of a multigene deletion system in *Lactiplantibacillus plantarum* with 99.4% correctness in the colonies analyzed.^53^ Additionally, dsDNA recombination mediated by λ-Red and RecE/T systems can effectively improve the gene editing efficiency. The use of the conditionally lethal gene *pheS*∗ as a negative selection marker combined with the temperature-sensitive plasmid pGhost9 in *Lactobacillus lactis* and *Lacticaseibacillus casei* enabled rapid screening for *Lactobacillus* mutants, and dramatically improved the accuracy of gene editing.^110^ However, excision of the selection markers used in the dsDNA recombination process usually leaves scars.^111^ In order to make only necessary changes in the bacterial genome without additional antibiotic selection, the application of ssDNA recombination technology assisted by ssDNA binding proteins such as RecT or Bet in *Lactococcus lactis* was developed. A 10-fold increase in the recombination efficiency of ssDNA can be achieved by increasing the concentration of oligonucleotides or by using phosphorothioate oligonucleotides.^112^ However, ssDNA recombination technology is primarily applicable to gene editing such as point mutations or short fragment deletions.^140^

CRISPR-Cas systems are commonly expressed in *Lactobacillus*. It has been demonstrated that CRISPR repeats can be detected in 59.7% of the 1,262 *Lactobacillus* genomes, with the type II-A CRISPR-Cas system being the most prevalent sub-type.^141^ In contrast, the exogenous CRISPR-Cas systems contain three key components: sgRNA, the DSB-mediating proteins, and homologous repair template.^142^^,^^143^ These facts facilitate CRISPR-Cas systems in the manipulation of probiotic genes and the interaction with the host. For example, seven mucin genes in *Lactiplantibacillus plantarum* AR113 were knocked out by CRISPR-Cas9-mediated gene editing technology to understand the mechanism of *Lactobacillus* adhesion and colonization in the host intestine. The colonization ability of *Lactobacillus* was significantly reduced after deleting AR113 mucin genes, especially after the reduction of intestinal flora using polyethylene glycol. With the assistance of CRISPR-Cas9-enabled gene knockout, this study proved that both the intestinal flora environment and AR113 mucin gene-regulated adhesion ability of *Lactobacillus* affect the colonization in the intestinal tract.^113^ *Lactobacillus* can secrete antimicrobial substances that inhibit the growth of pathogens. By knocking out genes such as the pyrimidine regulatory gene (PyrR) using CRISPR-Cas systems, a decrease in the production of pyrimidine analogs was found with a subsequent reduction in antimicrobial capacity. This demonstrates that *Lacticaseibacillus casei* inhibits the growth of pathogens by synthesis and secretion of pyrimidine analogs.^114^

In addition to discover the intrinsic property of *Lactobacillus*, gene editing technologies were applied for the production of a wide range of beneficial metabolites.^144^ N-acetylglucosamine (GlcNAc) is the precursor of glycosaminoglycans, which is critical for maintaining the function of healthy cartilage and joint tissue.^145^ CRISPR-Cas9 technology-assisted dsDNA and ssDNA recombination could enable *Lactiplantibacillus plantarum* WCFS1 to produce GlcNAc efficiently with a yield of up to 797.3 mg/L without introducing exogenous genes or plasmids. This facilitates the application of seamless gene editing technology in GlcNAc production.^115^

### EcN

EcN is the only non-pathogenic strain of *E. coli* isolated in 1917. It is capable to colonize the gastrointestinal tract, inhibit the invasion of pathogenic bacteria, and maintain the intestinal homeostasis. Therefore, EcN is a beneficial probiotic that can be used to treat inflammatory gastrointestinal dysfunctions.^146^^,^^147^ EcN is the active component of microbial drug Mutaflor for the treatment of gastrointestinal disorders including diarrhea, diverticular disease and ulcerative colitis.^148^ Moreover, EcN has excellent genetic stability and can be genetically modified with a variety of molecular biology tools for advanced preventive and therapeutic effects.^149^

I-SceI is a type I intron-encoded homing endonucleases, also known as meganuclease.^150^ Similar to restriction endonucleases, I-SceI is able to cleave the dsDNA in the presence of divalent metal ions with high specificity. This process generates DSBs at the target site to facilitate homologous recombination for gene insertion, deletion, or replacement.^151^ The gastrointestinal gene of interest (GOI) was integrated into a suicide plasmid carrying the I-SceI recognition site. GOI insertion at different sites in the EcN genome was implemented after the expression of the I-SceI meganuclease.^116^ EcN lacks the T7 RNA polymerase gene, which limits the use of expression vectors based on T7 promoters.^152^ The T7 RNA polymerase gene can be integrated into the *malEFG* operon of EcN by λ-Red recombination technology to construct a T7-promoter EcN strain, which can overcome the rate-limiting process of heme biosynthesis. This enabled EcN to efficiently produce heme proteins and heme-derived molecules.^117^ Heparosan is a polysaccharide precursor for the synthesis of the anticoagulant drug heparin. Multiple 19-kb *kps* sites can be inserted to EcN by RecA recombinant technology, and 9.1 g/L of heparosan can be produced by fermentation, making the industrial production of heparin through probiotics viable.^118^

The application of CRISPR-Cas9 system was able to efficiently and precisely remove the two cryptic plasmids from EcN to obtain the derivative strain EcNP to reduce the metabolic burden. Afterward, the *gadB* gene encoding glutamic acid decarboxylase was transfected into EcNP strain to produce gamma-aminobutyric acid (GABA) with high yield of 17.9 g/L, which can scale-up the industrial production for hypertension, epilepsy, anxiety disorders and other disease treatments.^119^ Using CRISPR-Cas9-edited EcN to produce desired products, such as for Parkinson’s disease (PD) or diabetes, has also been conducted. CRISPR-Cas9 system was used to integrate the gene encoding glucagon-like peptide (GLP-1), a therapeutic drug for maintaining blood glucose homeostasis, into the *attB* locus of the EcN genome to construct EcN-GLP-1. EcN-GLP-1 is capable of continuously secreting GLP-1 into the intestinal tract and regulating the balance of intestinal bacterial flora, thereby exerting a long-term therapeutic effect.^120^ In addition, the ability of EcN to colonize the mucosal surface and its strong immune-activating effects make it a versatile platform for the display of pathogenic antigens. Expression of HIV-1 periplasmic outer region (MPER) antigen by EcN using CRISPR-Cas9 system was applied for HIV vaccine study. Specifically, the EcN outer membrane protein OmpF was fused with MPER to construct stably transformed *EcN* strains. The final transformed EcN strains were capable of producing 14.3 μg/10^8^ cfu of MPER, providing a cost-effective platform for HIV vaccination.^121^

### Bacillus subtilis

*Bacillus subtilis* is a Gram-positive bacterium capable of forming spores and is highly resistant to specific conditions such as temperature, pH and bile salts, and thus can easily adapt to the environment in gastrointestinal tract.^153^ *Bacillus subtilis* is a Generally Recognized as Safe (GRAS) probiotic and plays an important role in the food and pharmaceutical industries for the production of enzymes, drug precursors, and a wide range of biologics.^154^ *Bacillus subtilis* is able to synthesize bacteriostatic agents in the gastrointestinal tract to inhibit the growth of pathogens while maintaining intestinal homeostasis.^155^

*Bacillus subtilis* is currently used as one of the model bacterium in the industry for the mass production of engineered products.^156^ The initial application of genetic engineering using λ-Red recombination system in *Bacillus subtilis* combined with Cre/*loxP* system was able to knockout of the *myc* gene cluster, while the editing efficiency remain low.^122^ To improve the efficiency, application of the homologous protein GP35 of RecT led to increase in the recombination frequency to 1.71 ± 0.15 × 10^−1^. However, this method relies on ssDNA and long homologous arms (500 bp), which is a time-consuming procedure.^123^ The use of 5′-phosphorothioate modified dsDNA as substrate in combination with the recombinase pair YqaJ/YqaK greatly improved the efficiency in knockout and knock-in in *Bacillus subtilis* 1A751.^124^ In order to enable the expression of natural product-derived gene clusters in *Bacillus subtilis*, two large gene fragments of the biosynthetic pathways from *Brevibacillus brevis* X23 and *Bacillus amyloliquefaciens* FZB42 were directly integrated into the genome of *Bacillus subtilis* using Red/ET recombination, resulting in the heterologous expression of large gene clusters.^125^

Traditional scarless genome engineering approaches are based on a selection-counterselection system and are often limited by the lack of suitable counterselection markers, the toxicity of the counterselection compounds, and the need to mutate the target strains.^157^ With the emerging potential of CRISPR-Cas9 system, gene editing in *Bacillus subtilis* become more feasible. For example, gene editing of *Bacillus subtilis* can be achieved by integrating the *cas9* gene controlled by the *Bacillus subtilis* mannose-inducible promoter P_*manP*_ and sgRNA sequences transcribed from the strong promoter in the shuttle plasmid pJOE8999.^126^ Since CRISPR-Cas9-induced DSBs lead to decreased bacterial survival, the efficiency of transformation and multiplex gene editing is low. dCas9 is a mutant form of the Cas9 protein. It has lost its shearing activity and can only be guided into the genome by sgRNA.^158^ Therefore, the base editing technology combining CRISPR-dCas9 system and cytidine deaminase was developed. Utilizing the ability of cytidine deaminase to mutate cytosine to thymine, early termination codons as well as targeted point mutations can be generated. The system enabled simultaneous gene editing on three or four loci with 100% and 50% efficiency, respectively.^32^ Compared to traditional Cas9-mediated gene editing methods, Cas9-nuclease (Cas9n)-mediated gene editing causes less damage and toxicity to the host, and single-strand breaks are easier to repair. CRISPR-Cas9n system is more efficient for large fragments editing as well as multiplex gene editing in *Bacillus subtilis*.^127^ Cas12a, known as Cpf1, stands as a distinguished member of the type V-A CRISPR effector family, serving as an RNA-guided DNA endonuclease. CRISPR-Cas12a (Cpf1) system enables double gene knockout, multipoint mutation or single gene insertion in *Bacillus subtilis* at the same time with high efficiency. These approaches promote the development of *Bacillus subtilis* in metabolic engineering.^128^

### Bifidobacteria

*Bifidobacteria* are Gram-positive bacteria that are commonly found in the oral cavity, gastrointestinal tract and vagina of mammals.^159^ Several *Bifidobacteria* species have been commercialized as probiotics due to the beneficial effects on human life and their intrinsic safety, such as *Bifidobacterium bifidum*,^160^ *Bifidobacterium breve*,^161^ and *Bifidobacterium longum*.^162^ However, *Bifidobacteria* are genetically recalcitrant, so developing universal gene editing tools for *Bifidobacteria* remains challenging.^163^

One successful example was the knockout and knock-in using suicide plasmids in *Bifidobacterium longum* engineering. By inducing recombinase expression of an excision replicon to cut the plasmid, homologous recombination is facilitated between the vector and the *Bifidobacterium longum* genome. This approach enables efficient gene editing independent of transformation efficiency.^129^ The genome of *Bifidobacteria* is enriched in CRISPR-Cas systems, but the editing of *Bifidobacteria* genes using this system has been less frequently reported.^164^ To apply CRISPR-Cas editing tools to genetically refractory *Bifidobacteria*, personalized editing strategies need to be designed for individual strains. Type I CRISPR system is the most common type of CRISPR in bacteria, of which type I-G is a subtype that has been relatively poorly characterized. It has been reported that drug-resistant *Bifidobacterium lactis* strains can be resensitized to tetracycline by using the endogenous type I-G CRISPR-Cas system in combination with an exogenous CRISPR base editor.^130^ However, more efficient gene-editing molecular biology techniques are desired for the application of *Bifidobacteria* in food and pharmaceutical engineering.

### Saccharomyces

*Saccharomyces* is a single-celled fungus that can ferment sugars into alcohol and carbon dioxide.^165^ In addition to being widely used in food and wine production, *Saccharomyces* can inhibit the proliferation of intestinal pathogenic bacteria, which can contribute to the balance of the intestinal microenvironment and enhance the host’s immune response.^166^^,^^167^ *Saccharomyces boulardii*^165^ and *Saccharomyces cerevisiae*^168^ have been found to be beneficial probiotics applied to the prevention and treatment of diseases.

*Saccharomyces* was one of the first microorganisms capable of gene editing by CRISPR-Cas systems.^169^ *Saccharomyces cerevisiae* has a high capacity for homologous recombination and can be used in conjunction with CRISPR-Cas systems to achieve efficient gene editing without the need for selection markers.^170^^,^^171^ Additionally, it has a lower incidence of off-targeting compared to other eukaryotic cells.^172^

*Saccharomyces cerevisiae* is a suitable organism for producing recombinant proteins. The δ-integration method can be used to integrate a high copy number of target genes into the yeast chromosome, enabling large-scale production of recombinant proteins.^173^ The CRISPR-δ integration method involves the pre-breakdown of the δ-sites on the yeast chromosome using CRISPR-Cas systems, followed by increasing the copy number of the integrated genes through the δ-integration, resulting in the activity of carboxymethyl cellulase (CMCase) to 559 U/L.^131^ Similarly, CRISPR-Cas systems were used to generate DSBs by cleaving at the δ-sites in *Saccharomyces cerevisiae*. A 24 kb DNA fragment containing the xylose utilization and (*R*, *R*)-2, 3-butanediol (BDO) production pathways was integrated into the yeast chromosome, resulting in the production of 12.51 g/L of BDO from 80 g/L of glucose.^132^

### Next-generation probiotics

Compared to traditional probiotics, gene editing of next-generation probiotics has been reported less frequently due to their infancy. Here we will discuss the gene editing technologies applied to *Akkermansia muciniphila*, *Bacteroides*, *Clostridium*, and *Eubacterium* (as shown in Table 4).

**Table 4:** List of next-generation probiotics for the gene editing technologies and application

Bacteria	Technologies	Features & Applications
*Akkermansia muciniphila*	Transposon mutants	Recognizing important genes such as MUL genes in repressing genes for cholesterol biosynthesis^174^
*Bacteroides*	Suicide plasmids	Constructing allelic deletions and substitutions for drug-resistant *Bacteroides* and counter-selection using the toxin Bfe1^175^
	CRISPR-mediated base editing tool, pnCasBS-CBE	Introducing non-synonymous mutations and stop codons into genes involved in carbohydrate metabolism in *B. theta*^176^
	CRISPR-Cas12a (Cpf1) system induced by aTc	Efficiently knocking down multi locus and large fragment (48 kb) genomes, as well as achieving high-efficient insertion of the exogenous gene *gfp* in *B. theta*^105^
	CRISPR-Cas12a (Cpf1) system	Investigating the role of the PULs in *Bacteroides uniformis* adaptation^177^
	aTc-induced Cas9-cytidine deaminase mutagenesis	Knocking down the abundance of human intestinal *Bacteroides* and applying cytidine deaminase mutagenesis to introduce a stop codon into the PUL genome to reveal the strategy to exploit polysaccharide^178^
	Site-specific double crossover mediated by NBU2 integrase	Increasing the maximum titer and specific productivity of butyrate^179^
	CRISPR-dCas9 system	Detecting bile acid and aTc in the human gut^180^
*Clostridium*	ClosTron	Investigating the effect of the β-hydroxybutyryl-CoA dehydrogenase gene on the metabolism of *Clostridium butyricum* and the function of *dlt* operon^181^^,^^182^
	Heterologous type II CRISPR-Cas9 system and endogenous type I-B CRISPR-Cas system	Enhancing butyrate production using highly efficient endogenous CRISPR-Cas in *Clostridium butyricum*^183^
	CRISPR-Cas12a (Cpf1) system	Achieving highly efficient and rapid genome modification and producing near-infrared fluorescence from biliverdin and hemin^184^
	CRISPR-Cas9 system	Elucidating clostridial synthesis of several different branched short-chain fatty acids^185^^,^^186^
	CRISPR-Cas9 system	Enabling the secretion of IL-2 for tumor immunotherapy^187^
*Eubacterium*	Suicide plasmids combined with aTc-induced counter-selection system	Allowing rapid, precise, antibiotic-label-free gene knockout in *Eubacterium*^188^
	CRISPR-EvCas9 system	Improving the knockdown efficiency^189^
	CRISPR-Cas9 system induced by aTc	Demonstrating that substantial repression of multiple genes in the Wood-Ljungdahl pathway and fructose-phosphotransferase system^190^

Abbreviations: MUL: mucin utilization locus, IL-2: interleukin-2, aTc: anhydrotetracycline, PULs: polysaccharide utilization sites.

### Akkermansia muciniphila

*Akkermansia muciniphila* are Gram-negative anaerobic bacteria that colonize the human gut throughout life, and typically constituting 1–4% of all bacteria in fecal samples from healthy adults. *Akkermansia muciniphila* utilize gastrointestinal mucin as their sole source of carbon and nitrogen, thereby effectively increasing mucus thickness and improving intestinal barrier function.^191^ *Akkermansia muciniphila* deficiency has been implicated in the development of a variety of diseases, including metabolic disorders,^192^ neurological disorders,^193^ infections^194^ and cancers,^195^ as well as aging.^196^

However, gene editing of *Akkermansia muciniphila* is challenging, in part because their sensitivity to oxygen and tiny colony morphology limit the engineering of *Akkermansia muciniphila*. In addition, the survival and proliferation of *Akkermansia muciniphila* in the gut depend on the metabolism of mucins, a specific physiological requirement that may make gene editing difficult under laboratory conditions.^197^ Raphael et al. developed the transposon insertion plasmid pSAM_Akk using the codon-optimized transposase Himar1c9, and constructed an *Akkermansia muciniphila* transposon mutation library by conjugation with the *E. coli* donor. The Tn mutants from the transposon library were grown in a restriction medium with gastric mucin as the sole carbon and nitrogen source and it was found by transposon sequencing that *Akkermansia muciniphila* was required to grow on mucin medium via *de novo* biosynthesis of amino acids. Moreover, *Akkermansia muciniphila* expressed the mucin utilization locus (MUL) genes to utilize mucin, allowing it to colonize the gut by competing with other microbes.^174^ This provides an essential tool for unraveling the molecular links between mucin metabolism, regulation of lipid homeostasis, and potential probiotic activity, as well as new opportunities for the future application of gene editing technologies in *Akkermansia muciniphila*. In 2022, Ouwerkerk et al. isolated six new strains of *Akkermansia muciniphila* from healthy humans and determined the presence of Cas1, Cas2, and Cas9/Csn1-encoding genes in *Akkermansia muciniphila*, which indicates that endogenous CRISPR-mediated gene editing in *Akkermansia muciniphila* also holds potential.^198^

### Bacteroides

*Bacteroides* are anaerobic, non-sporulating Gram-negative bacteria with a wide metabolic potential in the gastrointestinal microbiota, with the unique ability to digest a diverse range of carbohydrates and to produce short-chain fatty acids.^199^ *Bacteroides* can persistently colonize the gut by adapting its cell surface to the intestinal fluid environment because of dynamic changes in its surface structure.^200^^,^^201^ A variety of *Bacteroides* have been known to have the potential to act as probiotics with important roles in gut microbial communities and immune system maintenance, such as *Bacteroides thetaiotaomicron*,^202^ *B. vulgatus*^203^^,^ *B. fragilis*,^204^ and *B. uniformis*.^205^

Several gene editing technologies have been used for *Bacteroides* modification, including suicide plasmids,^206^ transposon mutagenesis,^207^ inducible CRISPRi and recombinase systems,^208^ CRISPR-Cas systems.^105^ Recent studies have shown that most *Bacteroides* are resistant to erythromycin and tetracycline. The researchers developed the inulin selection cassette as an alternative screening tool for drug-resistant *Bacteroides* by constructing a suicide plasmid to achieve allelic deletions and substitutions. The plasmid also allowed counterselection with the Bfe1 toxin from the secretion system of *Bacteroides fragilis* type VI without generating mutant background strains.^175^ To improve the efficiency of gene editing, Liang et al. developed a CRISPR-mediated base editing tool, pnCasBS-CBE, which successfully introduced non-synonymous mutations and stop codons into genes involved in carbohydrate metabolism in *B. thetaiotaomicron*, with editing efficiencies ranging from 15% to 100%. Zheng et al. discovered that CRISPR-Cas12a (Cpf1) system induced by anhydrotetracycline (aTc) could efficiently knockdown multi locus and large fragments (48 kb) in genomes, as well as achieve high-efficiency insertion of the exogenous gene *gfp* in *B. thetaiotaomicron*, with insertion efficiency of over 80%. Overall gene editing efficiency was over 60% and up to 100% efficiency in *B*. *vulgatus*.^105^

Gene editing of *Bacteroides* also leads to a better understanding of its physiological characteristics and mechanism of action. Targeted knockdown of polysaccharide utilization sites (PULs) in *B*. *uniformis* using CRISPR-Cas12a (Cpf1) system provides a deep understanding of how different PULs contribute to polysaccharide utilization, microbial interactions, and colonization of the mammalian gut.^177^ In addition, Beller et al. used aTc-induced Cas9-cytidine deaminase mutagenesis to reduce the abundance of human intestinal *Bacteroides* and applied cytidine deaminase mutagenesis to introduce a stop codon into the genome of PULs, revealing a strategy to exploit polysaccharide.^178^

In addition to these basic metabolic studies, gene editing of *Bacteroides* has been used to enhance the product yield and for biosensing. Metabolic engineering of *B. thetaiotaomicron* was predicted using genome-scale metabolic modeling and heterologous production of unnatural butyrate up to 12 mg/L was achieved by homologous recombination. The maximum titer and specific productivity of butyrate in the *pta-ldhD* double knockout mutant mediated by NBU2 integrase were increased by nearly 3.4-fold and 4.8-fold compared to wild-type, respectively.^179^ Automated CRISPR-dCas9-based genetic logic circuits designed within *B. thetaiotaomicron* as the chassis can be used in biosensing to detect bile acid and aTc in the human gut. The detection limits for isopropyl-β-D-1-thiogalactopyranoside, deoxycholic acid, and aTc are 500 μM, 62.5 μM, and 100 ng/μL, respectively.^180^

However, there are still a number of challenges associated with *Bacteroides* engineering, mainly related to the lack of understanding of the dynamics and functions of each strain and the mechanisms of their interactions with the host.^209^

### Clostridium

*Clostridium* is a Gram-positive, specialized anaerobic bacterium that colonizes the intestinal tract of humans and other animals.^210^ In an aerobic environment, *Clostridium* exists as inert endospores, and it germinates into metabolically active trophoblasts when oxygen is absent.^211^ *Clostridium* primarily utilizes indigestible polysaccharides and produces most of the metabolites that increase intestinal immune tolerance and reduce inflammation.^212^ In the *Clostridium* genus, *Clostridium butyricum*^213^ and *Clostridium sporogenes*^214^ are recognized as valuable probiotics with significant biological functions.

As mentioned above, ClosTron is a kind of group II introns developed for the transformation of *Clostridium*. Efficient gene knockout has been achieved in many *Clostridium butyricum* using ClosTron. These studies include exploring the effect of the β-hydroxybutyryl-CoA dehydrogenase gene on the metabolism of *Clostridium butyricum*,^181^ and finding that a functional *dlt* operon controls the d-alanylation of cytoderm components and affects cell septation and vancomycin-induced lysis.^182^ Moreover, the coupling efficiency of endogenous CRISPR-Cas systems has been shown to be much higher than that of CRISPR-Cas9 systems imported into *Clostridium*.^96^^,^^215^ Therefore, the chances of successfully establishing efficient genome editing tools are usually higher when using natural CRISPR-Cas mechanisms than when using heterologous CRISPR-Cas9 systems. Researchers applied the heterologous type II CRISPR-Cas9 system and the endogenous type I-B CRISPR-Cas system to perform double knockout in *Clostridium butyricum* to enhance butyrate production, and found that reusing the endogenous CRISPR-Cas mechanisms to genetically engineer *Clostridium butyricum* has numerous advantages, such as small vector, low toxicity, high efficiency, and slight off-targeting. The efficiency is as high as 100% compared to the heterologous type II CRISPR-Cas9 system.^183^ In order to expand CRISPR-Cas-mediated gene editing tools for *Clostridia*, CRISPR-Cas12a (Cpf1) system with two different *cas12a* genes (*Ascas12a* and *Fncas12a*) was established. It shows that CRISPR-Cas12a (Cpf1) system provides flexible target selection in *Clostridia* and the specific folding pattern of the precursor crRNA is important for achieving high mutation generation efficiency. The optimized donor DNA template for gene integration in CRISPR-Cas12a (Cpf1) system achieved highly efficient (85–100%) and rapid (∼1 week) gene modification and successfully engineered *Clostridia* (*Clostridium butyricum* and *Clostridium sporogenes*) to produce near-infrared fluorescence from biliverdin and hemin.^184^

*Clostridium sporogenes* was found valuable in tumor treatment.^216^ However, gene editing operations in *Clostridium sporogenes* are not straightforward. After struggling for over nine months with no live colonies, Guo et al. discovered that the sgRNA and Cas9 components must be introduced separately into *Clostridium sporogenes* to effectively promote extracellular DNA uptake and homologous recombination. They first successfully developed the CRISPR-Cas9 deletion system in *Clostridium sporogenes* in 2019, using deletion mutants and mass spectrometry to elucidate clostridial synthesis of several different branched short-chain fatty acids.^185^^,^^186^ Kubiak et al. adopted CRISPR-Cas9 system to delete the toxin manipulator in *Clostridium sporogenes* NCIMB 10696. This strain was programmed to secrete mouse-activated Interleukin-2 (IL-2) to stimulate T cell proliferation for tumor immunotherapy.^187^

### Eubacterium

*Eubacterium* is a Gram-positive bacterium, one of the core genera of the human gut microbiota. As a consensus probiotic among gut microbiologists, it has the ability to promote the maintenance of intestinal flora balance and inhibit intestinal inflammation.^217^

Researchers constructed an aTc-induced counterselection system using the RelB family of toxins in *Eubacterium callanderi*, which was combined with a non-replicating mutant vector to construct a suicide plasmid. The expression of RelB family toxins was induced by aTc, which resulted in the inhibition of cell growth. Only those that had lost the integrating plasmid and toxin genes through a second recombination event could be grown on aTc-containing medium, allowing for rapid, precise, and antibiotic-label-free gene knockout in *Eubacterium*^188^*.* Several novel Cas nucleases have been identified in *Eubacterium*. A compact type II-A *Eubacterium ventriosum* CRISPR-Cas9 system (EvCas9) was characterized from *Eubacterium ventriosum*. Compared with SpCas9 and SaCas9, EvCas9 has the advantages of simpler PAM, suitable size for AVV packaging, and dual-recognition mechanism, which was designed as an efficient base editor by fusing cytidine or adenosine deaminase. Knockdown efficiency in bacteria can reach 57–100% using CRISPR-EvCas9 system.^189^ The aTc-induced CRISPR-Cas9 system can precisely modify target genes with 100% efficiency. By introducing dCas9 protein, expression of the target gene can be effectively inhibited in *Eubacterium limosum*, and several genes in the Wood-Ljungdahl pathway and fructose-phosphotransferase system showed substantial inhibition greater than 84%.^190^

## Concluding remarks and future perspectives

Probiotics are usually provided in the form of natural products or nutritional agents to maintain human health. However, there is considerable variation in the tolerance and colonization abilities of probiotics, which constrains their applicability.^218^ Gene editing technologies can enhance the stability and functionality of natural probiotics, making engineered probiotics a promising candidate for development as biotherapeutics.^219^ Although there is increasing diversity and feasibility in the engineering refurbishment of probiotics, it is important to note that only a limited portion of the microbial community is currently accessible and suitable for gene editing. Numerous advantageous microorganisms exhibit limited accessibility to genetic tools, and the gene editing procedure often entails off-targeting, diminished transformation efficacy, and potential hazards in heritable editing. The modification and precise editing of large gene fragments in probiotics continue to provide significant challenges. The application of gene editing technology in probiotics presents several significant hurdles. (1) The precise identification and consistent existence of the target genes, as well as the continuous generation of the desired substances; (2) the regulation of the interaction between the modified probiotic and other microorganisms in the gastrointestinal tract; (3) the potential for delivery of gene editing toolbox to edited probiotics; and (4) the screening after transformation for the selection of desired strains.^220^ The process of base editing involves the integration of programmable DNA-binding proteins, such as Cas9, with base deaminase in order to achieve single-base DNA alterations. This integration has been shown to greatly enhance the effectiveness and precision of gene editing. The off-target effect of CRISPR-Cas systems is considerably reduced through the process of prime editing, which involves the extensive modification of Cas9 protein and gRNA.^221^ The application of omics technologies enables the identification of novel and effective probiotics within the human body.^219^ CRISPR-Cas systems can also facilitate the investigation of the relationship between probiotics and the host microbial environment when used in conjunction with omics technologies.^222^ RNA editing can serve as a supplementary method to DNA editing in probiotics in specific instances.^223^ One notable advantage lies in the brief life cycle of RNA, which allows for reversible and inheritable RNA modification. In cases where DNA-level variable editing of transcripts is not feasible, the sole means of modification is through RNA editing.

In comparison to the long-established practice of incorporating natural probiotics into food or dietary supplements, gene-edited probiotics warrant more rigorous regulation due to their synthetic modifications. The following factors should be taken into account during the regulatory process for engineered probiotics: (1) the provision of the gene sequence of the exogenously introduced gene to the regulatory agency; (2) the avoidance of the transfer of antibiotic resistance cassettes to the resident microbiota; (3) the assessment of the ability of the microorganisms to replicate or persist and to be cleared from the host and/or the environment; and (4) the observation of the biodistribution of the engineered microorganisms within the host.^224^ It is also imperative to prioritize the safety of the application process when utilizing probiotics. The integration of biocontainment systems within the genetic structure of probiotics represents a critical strategy for the prevention and/or control of the spread of these microorganisms into the environment.^225^

Through an examination of the reaction to therapeutic probiotics, significant disparities were observed in the host’s response across many genetic and environmental factors, as well as the considerable diversity within the host’s commensal microbial community. In the future, it is imperative to address the pre-existing imbalanced host microbial community and intestinal flora when genetically modifying probiotics for therapeutic purposes, particularly in the context of long-term disease therapies like irritable bowel disease and diabetes. The therapeutic efficacy of designed probiotics can be significantly influenced by endogenous variables, namely in relation to colonization, survivability, and functionality. The progress in automated machinery and gene editing technology has resulted in the development of diverse high-throughput screening techniques for reconstructing cell factories. These techniques involve altering the physiological functions of cells at the genomic level, thereby creating favorable circumstances for strain proliferation and product accumulation. Consequently, this enhances the production efficiency for personalized therapeutic interventions.

## Acknowledgments

This work was supported by grants from the 10.13039/501100012166National Key R&D Program of China (2022YFA1206100, 2021YFA1201100), 10.13039/501100004826Beijing Natural Science Foundation (Z230008), the 10.13039/501100001809National Natural Science Foundation of China (32271449, 32201158, 51773188), and CAS Project for Young Scientists in Basic Research (YSBR-036).

## Author contributions

L.W., J.H., and K.L. wrote the manuscript. Y.Z., and M.Z. revised the manuscript.

## Declaration of interests

The authors declare no competing interests.
